# 2,2′-(1,1′-Azinodiethyl­idyne)diphenol

**DOI:** 10.1107/S1600536808011318

**Published:** 2008-04-26

**Authors:** Xi-Shi Tai, Jun Xu, Yi-Min Feng, Zu-Pei Liang

**Affiliations:** a Department of Chemistry and Chemical Engineering, Weifang University, Weifang 261061, People’s Republic of China; b Weifang Institute of Supervision and Inspection of Product Quality, Weifang 261061, People’s Republic of China

## Abstract

In the title mol­ecule, C_16_H_16_N_2_O_2_, the C—N bond lengths are 1.295 (5) and 1.300 (5) Å, which suggests that they are double bonds. The structure is stabilized by intra­molecular O—H⋯N and C—H⋯N, and inter­molecular C—H⋯O hydrogen-bond inter­actions.

## Related literature

For related literature, see: Tai *et al.* (2003 ▶).
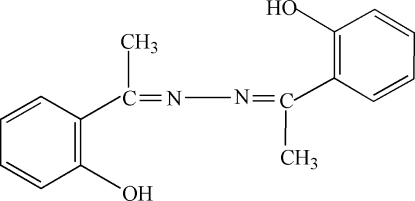


## Experimental

### 

#### Crystal data

C_16_H_16_N_2_O_2_
*M* *_r_* = 268.31
Orthorhombic, 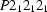

*a* = 6.3358 (8) Å
*b* = 13.5625 (10) Å
*c* = 15.9956 (15) Å
*V* = 1374.5 (2) Å^3^
*Z* = 4
Mo *K*α radiation
μ = 0.09 mm^−1^
*T* = 298 (2) K
0.38 × 0.15 × 0.14 mm

#### Data collection

Bruker SMART CCD area-detector diffractometer
Absorption correction: multi-scan (*SADABS*; Bruker, 2000 ▶) *T*_min_ = 0.968, *T*_max_ = 0.988
7170 measured reflections
1422 independent reflections
849 reflections with *I* > 2σ(*I*)
*R*_int_ = 0.044

#### Refinement

*R*[*F* ^2^ > 2σ(*F* ^2^)] = 0.042
*wR*(*F* ^2^) = 0.128
*S* = 1.08
1422 reflections
181 parameters
H-atom parameters constrained
Δρ_max_ = 0.15 e Å^−3^
Δρ_min_ = −0.14 e Å^−3^

### 

Data collection: *SMART* (Bruker, 2000 ▶); cell refinement: *SAINT* (Bruker, 2000 ▶); data reduction: *SAINT*; program(s) used to solve structure: *SHELXS97* (Sheldrick, 2008 ▶); program(s) used to refine structure: *SHELXL97* (Sheldrick, 2008 ▶); molecular graphics: *SHELXTL* (Sheldrick, 2008 ▶); software used to prepare material for publication: *SHELXTL*.

## Supplementary Material

Crystal structure: contains datablocks global, I. DOI: 10.1107/S1600536808011318/at2562sup1.cif

Structure factors: contains datablocks I. DOI: 10.1107/S1600536808011318/at2562Isup2.hkl

Additional supplementary materials:  crystallographic information; 3D view; checkCIF report

## Figures and Tables

**Table 1:** Hydrogen-bond geometry (Å, °)

*D*—H⋯*A*	*D*—H	H⋯*A*	*D*⋯*A*	*D*—H⋯*A*
O1—H1⋯N1	0.82	1.80	2.529 (5)	146
O2—H2⋯N2	0.82	1.80	2.529 (4)	147
C1—H1*A*⋯N2	0.96	2.32	2.739 (5)	106
C5—H5⋯O2^i^	0.93	2.59	3.403 (6)	147
C9—H9*A*⋯N1	0.96	2.30	2.724 (6)	106

Symmetry code: (i) 
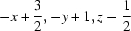
.

**Fig. 1.**
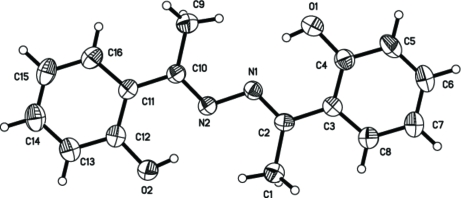
The molecular structure of (I) showing 30% displacement ellipsoids.

## supplementary crystallographic information

### Comment

As part of our ongoing studies of the coordination chemistry of Schiffbase ligands (Tai *et al.*, 2003), we now report the synthesis and structure of the title compound, (I), (Fig. 1).

In the molecule of (I), both C2—N1 [1.295 (5) Å], and C10—N2 [1.300 (5) Å] are close to double-bond separations, indicating that the Lewis structure shown in the scheme is only an approximation to the electron distribution in the molecule. Otherwise, the geometrical parameters for (I) are normal. The structure is stabilized by intramolecular O—H···N and C—H···N, and intermolecular C—H···O hydrogen bonding interactions.

### Experimental

2 mmol of 2'-Hhydroxyacetophenone (2 mmol) was added to a solution of hydrazide (1 mmol) in 10 ml of 95% ethanol. The mixture was continuously stirred for 3 h at refluxing temperature, evaporating some ethanol, then, upon cooling, the solid product was collected by filtration and dried *in vacuo* (yield 58%). Clear blocks of (I) were obtained by evaporation from a methanol solution after 6 days.

### Refinement

The H atoms were placed geometrically (C—H = 0.93–0.96 Å, O—H = 0.82 Å) and refined as riding with *U*_iso_(H) = 1.2*U*_eq_(aromatic C) or 1.5*U*_eq_(methyl C, hydroxyl O).

### Crystal data

**Table:**

C_16_H_16_N_2_O_2_	*F*_000_ = 568
*M**_r_* = 268.31	*D*_x_ = 1.297 Mg m^−^^3^
Orthorhombic, *P*2_1_2_1_2_1_	Mo *K*α radiation λ = 0.71073 Å
Hall symbol: P 2ac 2ab	Cell parameters from 1489 reflections
*a* = 6.3358 (8) Å	θ = 2.9–20.4º
*b* = 13.5625 (10) Å	µ = 0.09 mm^−^^1^
*c* = 15.9956 (15) Å	*T* = 298 (2) K
*V* = 1374.5 (2) Å^3^	Block, colourless
*Z* = 4	0.38 × 0.15 × 0.14 mm

### Data collection

**Table:**

Bruker SMART CCD area-detector diffractometer	1422 independent reflections
Radiation source: fine-focus sealed tube	849 reflections with *I* > 2σ(*I*)
Monochromator: graphite	*R*_int_ = 0.044
*T* = 298(2) K	θ_max_ = 25.0º
φ and ω scans	θ_min_ = 2.0º
Absorption correction: multi-scan(SADABS; Bruker, 2000)	*h* = −7→7
*T*_min_ = 0.968, *T*_max_ = 0.988	*k* = −16→13
7170 measured reflections	*l* = −17→19

### Refinement

**Table:**

Refinement on *F*^2^	Secondary atom site location: difference Fourier map
Least-squares matrix: full	Hydrogen site location: inferred from neighbouring sites
*R*[*F*^2^ > 2σ(*F*^2^)] = 0.042	H-atom parameters constrained
*wR*(*F*^2^) = 0.128	*w* = 1/[σ^2^(*F*_o_^2^) + (0.0468*P*)^2^ + 0.4138*P*] where *P* = (*F*_o_^2^ + 2*F*_c_^2^)/3
*S* = 1.08	(Δ/σ)_max_ < 0.001
1422 reflections	Δρ_max_ = 0.15 e Å^−^^3^
181 parameters	Δρ_min_ = −0.14 e Å^−^^3^
Primary atom site location: structure-invariant direct methods	Extinction correction: none

### Special details

**Table:**

Geometry. All e.s.d.'s (except the e.s.d. in the dihedral angle between two l.s. planes) are estimated using the full covariance matrix. The cell e.s.d.'s are taken into account individually in the estimation of e.s.d.'s in distances, angles and torsion angles; correlations between e.s.d.'s in cell parameters are only used when they are defined by crystal symmetry. An approximate (isotropic) treatment of cell e.s.d.'s is used for estimating e.s.d.'s involving l.s. planes.
Refinement. Refinement of *F*^2^ against ALL reflections. The weighted *R*-factor *wR* and goodness of fit *S* are based on *F*^2^, conventional *R*-factors *R* are based on *F*, with *F* set to zero for negative *F*^2^. The threshold expression of *F*^2^ > σ(*F*^2^) is used only for calculating *R*-factors(gt) *etc*. and is not relevant to the choice of reflections for refinement. *R*-factors based on *F*^2^ are statistically about twice as large as those based on *F*, and *R*- factors based on ALL data will be even larger.

### Fractional atomic coordinates and isotropic or equivalent isotropic displacement parameters (Å^2^)

**Table:**

	*x*	*y*	*z*	*U*_iso_*/*U*_eq_	
N1	0.5862 (6)	0.4828 (2)	0.5963 (2)	0.0489 (9)	
N2	0.4075 (6)	0.4685 (2)	0.6456 (2)	0.0486 (9)	
O1	0.8168 (6)	0.4367 (2)	0.47328 (19)	0.0824 (11)	
H1	0.7174	0.4320	0.5059	0.124*	
O2	0.1570 (5)	0.5228 (2)	0.75976 (17)	0.0670 (9)	
H2	0.2602	0.5259	0.7290	0.101*	
C1	0.6514 (8)	0.6288 (3)	0.6846 (3)	0.0719 (14)	
H1A	0.5090	0.6183	0.7031	0.108*	
H1B	0.6665	0.6953	0.6649	0.108*	
H1C	0.7468	0.6177	0.7302	0.108*	
C2	0.7010 (7)	0.5586 (3)	0.6150 (2)	0.0472 (10)	
C3	0.8877 (7)	0.5753 (3)	0.5630 (2)	0.0487 (11)	
C4	0.9357 (8)	0.5152 (3)	0.4941 (3)	0.0605 (13)	
C5	1.1111 (9)	0.5351 (4)	0.4453 (3)	0.0779 (16)	
H5	1.1417	0.4951	0.3996	0.093*	
C6	1.2395 (9)	0.6130 (4)	0.4636 (3)	0.0778 (15)	
H6	1.3570	0.6254	0.4304	0.093*	
C7	1.1973 (8)	0.6727 (4)	0.5301 (3)	0.0698 (14)	
H7	1.2848	0.7259	0.5422	0.084*	
C8	1.0243 (7)	0.6535 (3)	0.5790 (3)	0.0589 (12)	
H8	0.9973	0.6942	0.6246	0.071*	
C9	0.3515 (10)	0.3201 (3)	0.5599 (3)	0.0861 (17)	
H9A	0.4546	0.3497	0.5237	0.129*	
H9B	0.2270	0.3046	0.5283	0.129*	
H9C	0.4084	0.2608	0.5836	0.129*	
C10	0.2963 (7)	0.3905 (3)	0.6284 (2)	0.0502 (11)	
C11	0.1087 (7)	0.3741 (3)	0.6796 (3)	0.0500 (11)	
C12	0.0460 (7)	0.4402 (3)	0.7423 (3)	0.0542 (11)	
C13	−0.1329 (8)	0.4228 (4)	0.7893 (3)	0.0651 (13)	
H13	−0.1705	0.4669	0.8312	0.078*	
C14	−0.2557 (9)	0.3416 (4)	0.7750 (3)	0.0766 (15)	
H14	−0.3769	0.3310	0.8065	0.092*	
C15	−0.1990 (9)	0.2764 (3)	0.7141 (3)	0.0762 (15)	
H15	−0.2824	0.2212	0.7041	0.091*	
C16	−0.0201 (8)	0.2914 (3)	0.6674 (3)	0.0668 (13)	
H16	0.0165	0.2456	0.6267	0.080*	

### Atomic displacement parameters (Å^2^)

**Table:**

	*U*^11^	*U*^22^	*U*^33^	*U*^12^	*U*^13^	*U*^23^
N1	0.044 (2)	0.052 (2)	0.051 (2)	0.0052 (18)	0.0000 (18)	−0.0005 (16)
N2	0.046 (2)	0.047 (2)	0.053 (2)	0.0031 (18)	−0.0032 (18)	0.0000 (16)
O1	0.093 (3)	0.084 (2)	0.070 (2)	−0.013 (2)	0.021 (2)	−0.0175 (17)
O2	0.070 (2)	0.0691 (19)	0.0615 (19)	−0.0107 (19)	0.0074 (18)	−0.0083 (15)
C1	0.062 (3)	0.067 (3)	0.087 (3)	−0.006 (3)	0.012 (3)	−0.017 (3)
C2	0.049 (3)	0.044 (2)	0.049 (2)	0.010 (2)	−0.004 (2)	−0.0004 (18)
C3	0.048 (3)	0.052 (2)	0.047 (2)	0.009 (2)	−0.005 (2)	0.006 (2)
C4	0.068 (4)	0.062 (3)	0.052 (3)	−0.001 (3)	0.001 (3)	0.005 (2)
C5	0.087 (4)	0.092 (4)	0.054 (3)	0.005 (4)	0.020 (3)	0.004 (3)
C6	0.063 (3)	0.094 (4)	0.076 (4)	0.000 (3)	0.019 (3)	0.019 (3)
C7	0.056 (3)	0.076 (3)	0.076 (3)	−0.003 (3)	0.003 (3)	0.017 (3)
C8	0.053 (3)	0.063 (3)	0.061 (3)	0.003 (3)	−0.004 (3)	0.005 (2)
C9	0.079 (4)	0.072 (3)	0.107 (4)	−0.013 (3)	0.020 (3)	−0.035 (3)
C10	0.045 (3)	0.047 (2)	0.058 (3)	0.004 (2)	−0.004 (2)	−0.002 (2)
C11	0.049 (3)	0.045 (2)	0.056 (3)	0.001 (2)	−0.009 (2)	0.006 (2)
C12	0.055 (3)	0.054 (3)	0.054 (3)	0.001 (2)	−0.006 (2)	0.015 (2)
C13	0.063 (3)	0.070 (3)	0.063 (3)	0.005 (3)	0.002 (3)	0.015 (3)
C14	0.068 (3)	0.081 (3)	0.081 (4)	−0.002 (3)	0.009 (3)	0.031 (3)
C15	0.067 (4)	0.068 (3)	0.094 (4)	−0.019 (3)	−0.004 (3)	0.023 (3)
C16	0.068 (3)	0.057 (3)	0.075 (3)	−0.005 (3)	−0.009 (3)	0.003 (2)

### Geometric parameters (Å, °)

**Table:**

N1—C2	1.295 (5)	C7—C8	1.371 (6)
N1—N2	1.394 (4)	C7—H7	0.9300
N2—C10	1.300 (5)	C8—H8	0.9300
O1—C4	1.346 (5)	C9—C10	1.495 (5)
O1—H1	0.8200	C9—H9A	0.9600
O2—C12	1.352 (5)	C9—H9B	0.9600
O2—H2	0.8200	C9—H9C	0.9600
C1—C2	1.497 (5)	C10—C11	1.460 (6)
C1—H1A	0.9600	C11—C16	1.401 (5)
C1—H1B	0.9600	C11—C12	1.403 (6)
C1—H1C	0.9600	C12—C13	1.380 (6)
C2—C3	1.464 (5)	C13—C14	1.368 (6)
C3—C8	1.392 (5)	C13—H13	0.9300
C3—C4	1.405 (6)	C14—C15	1.364 (6)
C4—C5	1.385 (7)	C14—H14	0.9300
C5—C6	1.365 (7)	C15—C16	1.372 (7)
C5—H5	0.9300	C15—H15	0.9300
C6—C7	1.364 (6)	C16—H16	0.9300
C6—H6	0.9300		
			
C2—N1—N2	115.8 (3)	C7—C8—H8	118.8
C10—N2—N1	115.7 (3)	C3—C8—H8	118.8
C4—O1—H1	109.5	C10—C9—H9A	109.5
C12—O2—H2	109.5	C10—C9—H9B	109.5
C2—C1—H1A	109.5	H9A—C9—H9B	109.5
C2—C1—H1B	109.5	C10—C9—H9C	109.5
H1A—C1—H1B	109.5	H9A—C9—H9C	109.5
C2—C1—H1C	109.5	H9B—C9—H9C	109.5
H1A—C1—H1C	109.5	N2—C10—C11	116.5 (4)
H1B—C1—H1C	109.5	N2—C10—C9	123.2 (4)
N1—C2—C3	116.5 (4)	C11—C10—C9	120.3 (4)
N1—C2—C1	124.0 (4)	C16—C11—C12	116.5 (4)
C3—C2—C1	119.6 (4)	C16—C11—C10	121.2 (4)
C8—C3—C4	116.8 (4)	C12—C11—C10	122.3 (4)
C8—C3—C2	121.1 (4)	O2—C12—C13	117.1 (4)
C4—C3—C2	122.1 (4)	O2—C12—C11	122.0 (4)
O1—C4—C5	117.6 (4)	C13—C12—C11	120.9 (4)
O1—C4—C3	122.1 (4)	C14—C13—C12	120.9 (5)
C5—C4—C3	120.2 (5)	C14—C13—H13	119.5
C6—C5—C4	120.5 (5)	C12—C13—H13	119.5
C6—C5—H5	119.7	C15—C14—C13	119.5 (5)
C4—C5—H5	119.7	C15—C14—H14	120.3
C7—C6—C5	120.7 (5)	C13—C14—H14	120.3
C7—C6—H6	119.7	C14—C15—C16	120.6 (5)
C5—C6—H6	119.7	C14—C15—H15	119.7
C6—C7—C8	119.3 (5)	C16—C15—H15	119.7
C6—C7—H7	120.4	C15—C16—C11	121.7 (5)
C8—C7—H7	120.4	C15—C16—H16	119.2
C7—C8—C3	122.5 (5)	C11—C16—H16	119.2
			
C2—N1—N2—C10	−177.9 (4)	N1—N2—C10—C11	179.9 (3)
N2—N1—C2—C3	−179.2 (3)	N1—N2—C10—C9	−0.6 (6)
N2—N1—C2—C1	−0.2 (5)	N2—C10—C11—C16	−178.7 (4)
N1—C2—C3—C8	−178.6 (4)	C9—C10—C11—C16	1.7 (6)
C1—C2—C3—C8	2.3 (5)	N2—C10—C11—C12	2.2 (5)
N1—C2—C3—C4	2.7 (5)	C9—C10—C11—C12	−177.4 (4)
C1—C2—C3—C4	−176.4 (4)	C16—C11—C12—O2	−179.8 (4)
C8—C3—C4—O1	179.0 (4)	C10—C11—C12—O2	−0.7 (6)
C2—C3—C4—O1	−2.3 (6)	C16—C11—C12—C13	0.5 (6)
C8—C3—C4—C5	−0.3 (6)	C10—C11—C12—C13	179.6 (4)
C2—C3—C4—C5	178.4 (4)	O2—C12—C13—C14	179.2 (4)
O1—C4—C5—C6	−179.2 (4)	C11—C12—C13—C14	−1.1 (6)
C3—C4—C5—C6	0.1 (7)	C12—C13—C14—C15	0.8 (7)
C4—C5—C6—C7	−0.1 (8)	C13—C14—C15—C16	0.2 (7)
C5—C6—C7—C8	0.3 (7)	C14—C15—C16—C11	−0.8 (7)
C6—C7—C8—C3	−0.6 (6)	C12—C11—C16—C15	0.5 (6)
C4—C3—C8—C7	0.6 (6)	C10—C11—C16—C15	−178.7 (4)
C2—C3—C8—C7	−178.2 (4)		

### Hydrogen-bond geometry (Å, °)

**Table:**

*D*—H···*A*	*D*—H	H···*A*	*D*···*A*	*D*—H···*A*
O1—H1···N1	0.82	1.80	2.529 (5)	146
O2—H2···N2	0.82	1.80	2.529 (4)	147
C1—H1A···N2	0.96	2.32	2.739 (5)	106
C5—H5···O2^i^	0.93	2.59	3.403 (6)	147
C9—H9A···N1	0.96	2.30	2.724 (6)	106

Symmetry codes: (i) −*x*+3/2, −*y*+1, *z*−1/2.
